# A multicentre study on the clinical characteristics of newborns infected with coronavirus disease 2019 during the omicron wave

**DOI:** 10.3389/fped.2023.1192268

**Published:** 2023-07-17

**Authors:** Yi-Heng Dai, Caihuan Li, Guilong Yuan, Wenhui Mo, Jun Chen, Runzhong Huang, Zhonghe Wan, Duohua Lin, Xiangming Zhong, Huanqiong Li, Ling Liu, Jipeng Shi

**Affiliations:** ^1^ Department of Neonatal, Affiliated Foshan Maternity & Child Healthcare Hospital, Southern Medical University (Foshan Maternity &Child Healthcare Hospital), Foshan, China; ^2^ Department of Neonatal, Shunde Hospital of Southern Medical University, Foshan, China; ^3^ Department of Neonatal, Nanhai Maternity & Child Healthcare Hospital of Foshan, Foshan, China; ^4^ Department of Neonatal, Foshan Fosun Chancheng Hospital, Foshan, China; ^5^ Department of Neonatal, Shunde Women’s and Children’s Hospital of Guangdong Medical University, Foshan, China; ^6^ Department of Neonatal, The Sixth Affiliated Hospital of South China University of Technology, Foshan, China; ^7^ Department of Neonatal, Foshan Gaoming District People’s Hospital, Foshan, China; ^8^ Department of Neonatal, Sanshui Maternal and Child Health Hospital of Foshan City, Foshan, China; ^9^ Department of Neonatal, Sanshui District People’s Hospital of Foshan, Foshan, China; ^10^ Department of Neonatal, The Third Affiliated Hospital of Guangdong Medical University, Foshan, China

**Keywords:** neonates, coronavirus disease 2019, omicron wave, clinical features, routine blood tests

## Abstract

**Objective:**

To investigate the clinical characteristics and outcomes of newborns infected with coronavirus disease 2019 (COVID-19) during the Omicron wave.

**Methods:**

From December 1, 2022, to January 4, 2023, clinical data were collected from neonates with COVID-19 who were admitted to 10 hospitals in Foshan City, China. Their epidemiological histories, clinical manifestations and outcomes were analysed. The neonates were divided into symptomatic and asymptomatic groups. The *t* test or *χ*^2^ test was used for comparisons between groups.

**Results:**

A total of 286 children were diagnosed, including 166 males, 120 females, 273 full-term infants and 13 premature infants. They were 5.5 (0–30) days old on average when they were admitted to the hospital. These children had contact with patients who tested positive for COVID-19 and were infected through horizontal transmission. This study included 33 asymptomatic and 253 symptomatic patients, among whom 143 were diagnosed with upper respiratory tract infections and 110 were diagnosed with pneumonia. There were no severe or critical patients. Fever (220 patients) was the most common clinical manifestation, with a duration of 1.1 (1–6) days. The next most common clinical manifestations were cough with nasal congestion or runny nose (4 patients), cough (34 patients), poor appetite (7 patients), shortness of breath (15 patients), and poor general status (1 patient). There were no significant abnormalities in routine blood tests among the neonates infected with COVID-19 except for mononucleosis. However, compared with the asymptomatic group, in the symptomatic group, the leukocyte and neutrophil granulocyte counts were significantly decreased, and the monocyte count was significantly increased. C-reactive protein (CRP) levels were significantly increased (≥10 mg/L) in 9 patients. Myocardial enzyme, liver function, kidney function and other tests showed no obvious abnormalities.

**Conclusions:**

In this study, neonates infected with the Omicron variant were asymptomatic or had mild disease. Symptomatic patients had lower leucocyte and neutrophil levels than asymptomatic patients.

## Introduction

The coronavirus disease 2019 (COVID-19) pandemic that started in 2019 spread worldwide, and the COVID-19 epidemic in China is still ongoing. In particular, at the end of 2022, the Omicron variant spread to mainland China. The rapid spread of COVID-10 posed a great threat to a vast number of adults, children and newborns (1, 2). Some studies have shown that infection with COVID-19 is not commonly observed in hospitalized newborns (3). The majority of newborns have mild clinical manifestations and only require short-term hospitalization for treatment and most newborns present with symptoms of fever, upper respiratory tract infections, and lower respiratory tract infections (4). Multiple studies from different countries and regions and a meta-analysis have shown that the typical clinical manifestations of children with COVID-19 infection are fever, cough, and shortness of breath (1, 2, 5). Some studies have shown that common symptoms in newborns after contracting COVID-19 include shortness of breath and fever, and most newborns who are infected with the virus are asymptomatic or have mild symptoms and do not require respiratory support (6, 7). Another study showed that the most common symptoms observed in newborns infected with COVID-19 were fever, feeding intolerance, and cough, with myocarditis being the most common complication in those with severe-critical illness (8).

Previous studies showed that after COVID-19 infection, newborns generally had only mild clinical manifestations and did not need special treatment (9, 10). Nevertheless, certain studies have revealed severe scenarios. For instance, a study conducted in Brazil revealed that neonates who contracted COVID-19 during the initial phase of the pandemic experienced more severe clinical manifestations, which led to higher mortality rates. Such newborns displayed accelerated disease progression, longer hospital stays, and more pronounced respiratory distress, laryngospasm, and cough (11). Laboratory tests indicated a normal range of white blood cells and reduced lymphocyte levels (1, 2, 5, 6). However, newborns often have abnormal test results and imaging findings, including positive results for neutropenia and/or mononucleosis but not lymphocytopenia (12). There are also studies reporting that after infection with COVID-19, a reduction in platelet, lymphocyte, haemoglobin, eosinophil, and basophil counts and an increase in the neutrophil count, neutrophil-to-lymphocyte ratio, and platelet-to-lymphocyte ratio were commonly observed, and these indicators were associated with clinical prognosis (13, 14).

The clinical presentation and severity of COVID-19 have changed with the emergence of the Alpha, Beta, Delta and Omicron SARS-CoV-2 variants in different infection waves. Most published studies of neonatal COVID-19 infection were conducted before the Omicron wave. There is limited information on the clinical characteristics of neonatal COVID-19 infection caused by the Omicron variant. In this study, the data of neonates with COVID-19 who were admitted to 10 hospitals in Foshan from December 1, 2022, to January 4, 2023, were collected and analysed to investigate clinical characteristics and outcomes to provide some reference for the prevention and management of Omicron among newborns during the epidemic.

## Methods

### Study design and population

During the Omicron variant wave, it is recommended that all hospitalized newborns undergo routine COVID-19 testing. Furthermore, if a mother exhibits any symptoms of a respiratory tract infection, such as fever or cough, her newborn must be closely monitored in the maternal-child ward and undergo COVID-19 testing. Between December 1, 2022, and January 4, 2023, 286 neonates infected with COVID-19 were admitted to 10 hospitals. These newborns infected with COVID-19 were in the same room as their mothers after birth. If these newborns develop respiratory symptoms such as cough and fever, they should be admitted to the neonatology department for further observation. If a mother has symptoms of respiratory tract infection or has a history of contact with COVID-19-positive patients, the mother needs to take protective measures such as wearing a mask and washing hands before contact with children.

The inclusion criteria were as follows: (1) full-term infant age <28 days, preterm infant corrected gestational age <40 weeks; (2) pharyngeal swab results of COVID-19 nucleic acid test were positive or antigen-positive. Ethics approval was obtained from Foshan Maternity and Child Healthcare Hospital (approval number: FSFY-MEC-2023-022) in accordance with the Declaration of Helsinki.

There were a total of 13 premature infants included in this study. Any infant whose corrected gestational age exceeded 40 weeks did not meet our admission criteria for the neonatology department.

### Pharyngeal swab test for COVID-19

Pharyngeal swab samples were analysed at COVID-19 laboratories in several participating hospitals. The main kit used was the novel coronavirus pneumonia Nucleic Acid Detection Kit of Wuhan Mingde Biotechnology Co., Ltd. (National Instrument Note 20203400212), which uses PCR-fluorescence probe technology for detection. The procedures were performed according to the technical specifications and quality control specifications of the China National Clinical Laboratory Center.

Antigens in throat swab samples were detected by the Novel Coronavirus (2019-nCoV) Antigen Detection Kit (colloidal gold method) of Xiamen Aode Biotechnology Co., Ltd. The results are analysed as follows: if two dark or light red or purple bands appear, one in the testing area (T) and the other in the quality control area (C), this indicates a positive result. If only one red or purple band appears in the quality control area (C) and no band appears in the detection area (T), this indicates a negative result. If there is no red or purple band in the quality control area (C), regardless of whether there is a band in the detection area (T), the result is invalid, and retesting is necessary.

### Clinical data collection

The hospitalized neonates were registered, and information such as sex, age, gestational age, birth weight, epidemiological history, clinical symptoms, laboratory results, imaging results, treatment and length of stay were collected, and a clinical database was established.

### Clinical diagnosis and classification

The COVID-19 Pneumonia (Trial Version 9) criteria issued by the National Health Commission are used for the diagnosis and classification of cases (15). Asymptomatic infection was defined as those whose nucleic acid test met the diagnostic criteria but without any symptoms or signs; mild infection was defined as only mild clinical manifestations without imaging manifestations of pneumonia. Clinical and imaging manifestations of pneumonia were considered signs of general infection. Severe infection was defined as persistent high fever for more than 3 days, shortness of breath, hypoxemia, dyspnoea, lethargy, convulsion, food resistance or feeding difficulty with significant imaging findings of pulmonary inflammation. A critical infection was defined as secondary respiratory failure requiring respiratory support, shock, or a combination of other organ failures. The date when symptoms of COVID-19 infection appeared and the nucleic acid or COVID-19 antigen result was positive was considered the onset date.

### Discharge criteria

According to the discharge conditions stated in the Perinatal and Neonatal COVID-19 Infection Prevention and Control Plan (third edition), patients could be discharged once the patient's condition was stable, vital signs such as respiration and body temperature were normal, feeding tolerance was achieved, the family could provide reasonable care, and the discharge criteria were met (16, 17).

### Statistical analysis

We used SPSS 20.0 for statistical analysis, and measurement data are expressed as *x̅* ± *S*, minimum values and maximum value if necessary. Comparisons between groups were performed by *t* test or *t*'. The chi-square test or Fisher's exact probability method was used to compare the groups based on the statistical data. A bilateral value of *P *< 0.05 was considered statistically significant.

## Results

Most of the 286 patients had a contact history with family members who were positive for COVID-19. There were 166 male and 120 female patients, including 13 premature infants. Of the 286 newborns infected with COVID-19, 39 patients lacked data on amniotic fluid status. These infants had an average gestational age of 38.7 (29–41) weeks and an average birth weight of 2,949.4 ± 971.9 g. In total, 97 of the newborns were delivered by caesarean section, and 189 patients were delivered naturally. At admission, the average age of the patients was 5.5 (0–30) days. Among them, 134 newborns were exclusively breastfed, 14 newborns were fed formula, and 138 newborns received mixed feeding, as shown in Table 1.

**Table 1:** Baseline characteristics of neonates infected with COVID-19.

Variables	Asymptomatic group (*n* = 33)	Symptomatic group (*n* = 253)	*t* or *χ*^2^	*P*
Gender				
Male	22 (66.7)	144 (56.9)	1.14	0.286
Female	11 (33.3)	109 (43.1)		
Gestational age	38.1 ± 2.0	38.6 ± 1.3	−1.48	0.160
Birth weight	3,005.0 ± 864.5	3,143.0 ± 637.1	−1.06	0.289
Mode of delivery				
Spontaneous delivery	23 (69.7)	166 (65.6)	0.22	0.641
Caesarean section	10 (30.3)	87 (34.4)		
Amniotic fluid				
Clear	22 (71)	186 (86.1)	12.62	0.013
Maternal risk factors	22 (66.7)	156 (60.9)	3.22	0.2
Yes	10 (30.3)	100 (39.1)		
Feeding pattern				
Breast-feeding	17 (51.5)	117 (46.2)	5.25	0.073
Formula feeding	4 (12.1)	10 (4)		
Mixed feeding	12 (36.4)	126 (49.8)		
History of COVID-19 exposure	4 (12.5)	25 (10.3)	0.15	0.702
Yes	28 (87.5)	218 (89.7)		
Oxygen required	33 (100)	231 (91.3)	3.11	0.078
Yes	0	22 (8.7)		

Chest x-ray was performed for 227 patients; 64 patients showed no abnormality or thickened texture, 6 patients showed bronchitis-like changes, 157 (110 patients diagnosed with pneumonia and 47 who did not meet the diagnostic criteria for pneumonia) patients showed pneumonia-like changes, and 2 were diagnosed with pneumonia by chest CT examination. All children were given routine symptomatic treatment after admission and were discharged after their condition improved. The median length of stay was 5.6 (2–13) days.

### Clinical manifestations

Patients with fever, cough, nasal congestion or gastrointestinal symptoms were all regarded as symptomatic infected persons. There were 253 (88.5%) newborns with symptoms (Table 2). Among the children with symptomatic infection, 143 patients had mild infection, and 110 patients had common infections, such as fever (220 cases), cough with nasal congestion or runny nose (4 cases), cough (34 cases), poor appetite (7 cases), shortness of breath (15 cases), and poor general status (1 case). Fever was the most common clinical manifestation of COVID-19 infection, with fever occurring in 220 patients (76.9%), while 66 patients (23.1%) did not have a fever. Of those with fever, 181 had symptoms of fever alone. Some newborns exhibited noticeable respiratory symptoms, such as rapid breathing and breathing difficulties, necessitating respiratory support such as mechanical ventilation or noninvasive ventilation with a breathing machine, as shown in Table 2. No deaths occurred among these infants.

**Table 2:** Clinical symptom, treatment and short-term outcomes.

Variables	Number of cases or days	Percentage (%)
Fever and other symptom	220	76.9
Fever only	181	63.3
Cough	34	11.9
Shortness of breath	15	5.24
Poor appetite	7	2.4
Cough with nasal congestion or runny nose	4	14.0
Poor general status	1	0.3
Oxygen need	20	7
Non-invasive ventilator	3	0.1
Invasive mechanical ventilation	1	0.3
Length of Hospital stay	5.6	-

### Blood tests

The total number of white blood cells (9.7 ± 4.6) × 10^9^/L in the neonates infected with COVID-19 was within the normal range. However, the total number of leukocytes in symptomatic neonates was lower than that in asymptomatic neonates (9.4 ± 4.6 vs. 11.7 ± 4.2) × 10^9^/L), and the absolute value of neutrophils in the symptomatic neonates was lower than that in the asymptomatic neonates (3.7 ± 2.5 vs. 6.1 ± 3.6) × 10^9^/L). Neonates infected with COVID-19 had significantly higher monocyte counts (2.2 ± 1.5) × 10^9^/L than normal neonates. The absolute monocyte count was significantly higher in symptomatic neonates (2.2 ± 1.5) × 10^9^/L than in asymptomatic neonates (1.7 ± 1.1) × 10^9^/L. The percentage of monocytes was also significantly higher in the symptomatic group than in the asymptomatic group (22.8 ± 7.3 vs. 15.4 ± 9.1) × 10^9^/L, and the differences were statistically significant (*P* < 0.05).

The procalcitonin (PCT) level of neonates infected with COVID-19 was 0.9 ± 6.5 ng/ml, which was within the normal range. The CRP levels were normal (3.4 ± 9.7 mg/L) in the majority of patients, and only 9 patients had a significant increase in CRP levels (≥10 mg/L), suggesting the possibility of bacterial infection due to elevated levels of CRP. However, the blood culture results for these patients were negative. Myocardial enzyme CK-MB, alanine aminotransferase, aspartate aminotransferase, uric acid, urea, creatinine and other indexes in blood biochemistry were all within the normal range, as shown in Table 3.

**Table 3:** Laboratory test results.

Variables	Mean value	Standard deviation	Minimum value, Maximum value	Mean value	Standard deviation	Minimum value, Maximum value	*t*	*P*
	Asymptomatic group (*n* = 33)			Symptomatic group (*n* = 253)				
White blood cells	11.7	4.2	5.3, 19.8	9.4	4.6	3,26.1	2.77	0.010
Neutrophil count	6.1	3.6	0.8, 12.9	3.7	2.5	0.6, 15.5	4.79	<0.001
Neutrophil count %	49.8	17.6	5.8, 74.6	37.9	13.7	8, 75.1	4.54	<0.001
Monocyte	1.7	1.1	0.2, 5.8	2.2	1.5	0.2, 8.4	−1.97	0.050
Monocyte %	15.4	9.1	1.5, 36.2	22.8	7.3	2.8, 39.4	5.28	<0.001
Lymphocyte	3.6	2.2	1, 11.7	3.3	1.8	0.6, 9.7	0.99	0.320
Lymphocyte %	31.2	15.6	13.6, 83.9	36.3	14.6	9.6, 78	1.89	0.06
Red blood cell	4.6	0.6	3.2, 6.1	4.2	0.7	2.6, 7	3.4	<0.001
RBC volume distribution	55.1	9.1	16.1, 74.1	48.9	13.9	0.1, 72.2	2.49	0.010
PLT distribution	19.5	40.3	9.3, 240	16.7	38.0	8.6, 488	0.4	0.690
Average PLT volume	10.4	1.5	8.6, 15.2	11.0	1.5	7.9, 16.3	2.38	0.020
Hemoglobin	159.3	22.1	108, 209	137.7	23.4	33.7, 198	5.01	<0.001
Haematokrit	4.6	13.2	0.3, 48.5	4.4	12.4	0.3, 56.6	0.1	0.920
Mean RBC volume	99.0	7.2	76.9, 115.2	96.4	6.4	66.4, 113.4	−2.15	0.030
Platelets	281.0	90.8	108, 461	321.0	101.9	40, 658	2.15	0.030
C reactive protein	3.7	6.3	0, 29.2	3.3	10.1	0, 144.5	0.19	0.850
Procalcitonin	0.5	0.9	0.1, 3.9	0.9	6.9	0.1, 96.6	−0.28	0.780
Lactic dehydrogenase	485.7	256.0	273, 1,346	336.1	112.9	25, 1,116	4.15	<0.001
Creatine kinase	282.8	193.2	67, 828	130.7	66.8	34, 431	6.84	<0.001
Creatine kinase-MB	39.0	22.8	13.5, 110	25.4	30.9	0.3, 291	1.95	0.050
Beta-2 microglobulin	5.0	2.2	2.7, 9.9	12.7	56.5	2.5, 518	−0.45	0.650
Creatinine	44.4	19.6	11, 90	27.2	10.3	4.2, 84	7.79	<0.001
Uric acid	180.6	102.6	17, 460	161.4	58.8	34, 473	1.54	0.120
Blood urea	2.9	1.6	0.9, 8.4	3.2	2.1	0.7, 29	−0.81	0.420
Albumin	36.6	4.5	29.8, 56.5	36.9	3.0	29.9, 52.1	−0.63	0.530
Prealbumin	80.1	33.5	0.1, 139	69.0	40.3	0, 153	0.96	0.340
Alanine Aminotransferase	13.9	9.3	6, 55.9	19.0	13.0	4, 160	−2.13	0.030
Aspartate amino transferase	44.7	42.7	24.3, 233	39.5	20.7	1.7, 226	1.01	0.310
PH value	7.4	0.1	7.3, 7.5	7.4	0.1	7.3, 7.6	−1.52	0.130
Oxygen partial pressure	81.2	21.0	38.6, 109	79.7	23.4	23, 190	0.24	0.810
Pressure of carbon dioxide	31.9	6.6	21.8, 42.3	36.0	7.7	15.9, 78.7	−2.07	0.040
Lactic acid	2.7	1.4	0.8, 4.9	2.1	2.5	0.3, 30	0.89	0.370
Blood sugar	4.1	1.5	2.2, 7.6	5.2	1.3	2.8, 11.2	−2.78	0.010

Normal reference range of blood indexes: white blood cells: 15–20 × 10^9^/L, neutrophil count: 0.6–7.5 × 10^9^/L, monocyte: 0.15–1.56 × 10^9^/L, lymphocyte: 2.4–9.5 × 10^9^/L, red blood cell: 3.3–5.2 × 10^12^/L, mean RBC volume: 73–104 fL, red cell distribution width: 36–49 fL, PLT distribution 9–17 fL, average PLT volume: 9–13 fL, hemoglobin: 150–220 g/L, platelets: 100–300 × 10^12^/L, C reactive protein: <10 mg/L, LDH: 100–240 U/L, CK: 26–140 U/L, CK-MB: <50 U/L, beta-2 microglobulin: 1.01–2.97 mg/L, creatininer: 13–33 µmol/L, uric acid: 208–428 µmol/L, blood urea: 0.8–5.3 mmol/L, albumin: 35–55 g/L; prealbumin: 150–400 mg/L, ALT: <45 U/L, AST: <45 U/L; PO_2_: 50–80 mmHg, PCO_2_: 35–45 mmHg, LAC: <2.8 mmol/L, blood sugar: 2.66–7 mmol/L.

### Treatment and outcomes

A total of 286 children were admitted to the hospital after routine symptomatic treatment, and they were discharged after their condition improved. The average duration of hospitalization for neonates infected with COVID-19 was 5.6 (2.1–13) days.

In the neonatology department, some treatments were routinely given. Newborns with fever were typically managed with physical cooling methods, such as the application of ice pillows or reduction of the temperature of warm boxes. The use of drugs such as ibuprofen to lower fever in newborns was uncommon. When newborns experienced cough symptoms, they were often treated with a nebulized budesonide suspension, while oral ambroxol hydrochloride expectorant medication was recommended for newborns with phlegm. However, importantly, this treatment approach may not be considered completely standardized.

A total of 109 patients were treated with antibiotics prophylactically upon admission, the infection index became normal within 3 days, and the antibiotics were stopped after bacterial infection was excluded. No definitive evidence of bacterial infection was found in newborns with COVID-19 infection. In the study, prophylactic antibiotics were used for newborns with fever if bacterial infections could not be ruled out for some time. The commonly used drugs were ampicillin (50 mg/kg, q12h or q8h, depending on gestational age and postnatal age), piperacillin tazobactam (100 mg/kg, q12h or q8h), ceftazidime (50 mg/kg, q12h or q8h) and cefotaxime (50 mg/kg, q12h or q8h).

Some newborns and premature infants in this study were fed by nasogastric feeding due to their need for respiratory support or poor sucking ability. Once these newborns were weaned off mechanical ventilation or had developed a stronger suckling ability, the nasogastric tube was removed and replaced with self-suction.

## Discussion

During the COVID-19 pandemic, numerous published studies on COVID-19 infection in children, including newborns, were conducted prior to the Omicron variant outbreak. Because they have generally low immune function, newborns are more likely to suffer from COVID-19, so they need more attention. COVID-19 has mutated several times. The Omicron variant has become widespread, and the World Health Organization has defined it as the fifth variant of concern (18, 19). In a systematic review that included all articles published from December 1, 2019, to May 12, 2020, a quarter of newborns were asymptomatic, and the rest showed typical acute respiratory infections and/or gastrointestinal symptoms. Most did not need oxygen support, their average length of hospital stay was 10 days, and their prognoses were good (20). However, there have been few reports of newborn infections during the Omicron epidemic, and the clinical characteristics and prognoses of these newborns are not very clear.

The Foshan outbreak was caused by a variant of Omicron, which infected the vast majority of the population. The clinical symptoms in children after infection are not the exact same as those in adults (21). The main manifestations in children are fever, cough, sputum, nasal congestion, runny nose, headache, diarrhoea, abdominal distension, and anorexia, and some severe patients may have convulsion (5, 22). There have been scattered reports of COVID-19 infections among newborns in China (23, 24). The data are limited, and epidemiological investigations and clinical case analyses are lacking. In contrast, in most cohort studies of neonates, mild symptoms were reported, with common symptoms including shortness of breath, respiratory distress, fever, and symptoms related to gastrointestinal disorders (10, 25). In this study, the vast majority of newborns were in the same room as their mother and were breastfed. Mothers who have been infected with COVID-19 or those who have come into contact with COVID-19 patients routinely take some protective measures. There was no clear evidence of vertical transmission, which is consistent with other research results (26).

This study found that the vast majority of neonates infected with the Omicron variant showed symptoms, which is inconsistent with previous studies suggesting that most neonates infected with COVID-19 were asymptomatic and had mild symptoms (6, 7). A total of 110 neonates were diagnosed with pneumonia, and no severe or critical cases were observed. However, it should be noted that the diagnosis of pneumonia in this study may have been too broad, and the clinical manifestations were not serious, so the prognosis was not significantly different from that of neonates infected with upper respiratory tract infection. Notably, according to the clinical manifestations, chest radiographs and CT reports, a considerable number of infected neonates were diagnosed with pneumonia, but their clinical manifestations were mild. There was no significant difference in treatment or length of stay between the two groups.

In this study, fever was the most common clinical manifestation of COVID-19 infection, which is similar to many past studies (5). This was followed by cough, shortness of breath, poor appetite, cough with nasal congestion or runny nose, and poor spirit. The clinical manifestations of symptomatic neonates were generally not severe, and the duration of symptoms was not long, which is consistent with the reports of most previous studies (4, 5). Due to the limited long-term follow-up of these studies, current evidence cannot be used to conclude that there is no harm to neonates following infection with COVID-19. In this study, a few newborns with underlying diseases (such as persistent pulmonary hypertension, neonatal pneumonia, and malnutrition in premature infants) developed clinically unexplained severe conditions after infection with COVID-19, which resulted in a prolonged duration of severe symptoms (such as long-term mechanical ventilation, noninvasive ventilator-assisted ventilation, and oxygen inhalation) and hospital stay, but no serious complications were found in other infected children. A limitation of this study is that the diagnosis of neonatal pneumonia may have a problem with scope. In this study, the diagnosis of pneumonia was mainly based on clinical symptoms such as shortness of breath and cough plus chest radiograph or CT results because the symptoms of neonatal pneumonia are not typical.

There has not been much research on the laboratory testing of neonates infected with COVID-19, and these neonates may have normal or reduced white blood cell counts, a decreased neutrophil count that can persist for several months and/or lymphocytopenia (27–30). Previous studies have shown that newborns infected with COVID-19 have normal or decreased white blood cell counts and decreased lymphocyte levels compared with uninfected newborns (5, 6, 31). In contrast to previous studies, this study investigated the blood test results of symptomatic and asymptomatic newborns infected with COVID-19. In this study, the white blood cell counts and neutrophil levels were significantly decreased in neonates infected with COVID-19, but they were basically within the normal range, and these results were not entirely consistent with those reported in previous studies (1, 2, 5, 6). However, as previously reported, there was a significant decrease in the levels of leukocytes, neutrophils, and lymphocytes in newborns infected with COVID-19 who exhibited symptoms compared to those who were asymptomatic (29). This study had a larger patient sample size, and our research indicates that symptomatic neonates infected with the Omicron variant experience a significant decrease in the levels of neutrophils and lymphocytes.

Studies have shown that the monocyte levels of newborns infected with COVID-19 are increased (12). The proliferation of monocytes in this study was a noteworthy feature. Monocytes, a subset of white blood cells, mainly originate from myeloid progenitor cells in bone marrow, exist in the bloodstream and can differentiate into macrophages and dendritic cells (DCS) in tissues. As a result of pathological conditions, including viral infection, monocytes are activated and recruited by inflammatory mediators, migrate into affected tissues, and recruit macrophages and DC-like phenotypes. To realize the effector function of proinflammatory and anti-inflammatory activity, antigen presentation and tissue remodelling occur (32). They play an important role in host defence and excessive inflammation (19). Study results are inconsistent regarding changes in the number of monocytes in the blood during coronavirus infection. In some studies, flow cytometry analysis of blood samples from COVID-19 patients showed no change in the number of monocytes; however, the monocytes were larger than normal, which was related to the inflammatory phenotype (20). There are also studies showing that the number of monocytes in the blood of COVID-19 patients is significantly reduced (33). In the study by Andonegui-Elguera et al., the number of monocytes in the blood was increased, and the numbers of other cells, including lymphocytes, neutrophils, natural killer (NK) cells, and T cells, decreased significantly (34). In this study, it was found that the absolute value and percentage of monocytes increased significantly after COVID-19 infection, suggesting that monocytes may play an important role in neonates infected with COVID-19. Although mononucleosis is a double-edged sword, it does not cause serious cytokine storms in neonatal cases, its clinical symptoms are relatively mild, and its short-term prognosis is good.

Other indicators, such as CRP and PCT, in infected neonates were within the normal range, which was consistent with the typical characteristics of viral infection. According to previous studies, after adult infection with COVID-19, TNF-α, interleukin and other indicators have significant changes, and even some severely infected individuals have excessive inflammation and cytokine storm phenomena (34). Cytokines were not detected in this study, so further research is needed.

In previous studies, the transmission of COVID-19 was mostly horizontal, but the possibility of vertical transmission cannot be ruled out (24, 35). Maternal vaccination is a very important measure to prevent neonatal infection (36). Additionally, if the mother or other family members are infected with COVID-19, they should take protective measures, such as wearing a mask, washing their hands, and ventilating the room. Current research shows that if mothers take appropriate preventive measures, it is safe to allow newborns to be in the same room as their mothers and receive breast milk care directly (26). The main limitations of this study are the lack of information on the vaccination status of pregnant women and evidence of vertical transmission (e.g., COVID-19 status of mothers during delivery, placental examination, umbilical cord blood PCR, etc.). The other limitation of this study was the insufficient data available on maternal vaccination. This missing information could possibly provide insight into whether vaccinated mothers offer protection to their newborns.

In conclusion, newborns with COVID-19 infection caused by the omicron variant may be asymptomatic or have mild symptoms with a short duration and have a good short-term prognosis. However, long-term follow-up on physical and neurological development is still needed.

## Data Availability

The raw data supporting the conclusions of this article will be made available by the authors, without undue reservation.
